# Hydrometra

**Published:** 1850-11

**Authors:** 


*Hydrometra.* Case reported by Dr. D. H. Storer.

The patient was a large and very fleshy woman, thirty-five years of age, and weighed about 230 pounds. In June, 1849, I saw her for the first time, and received the following history: that she had been married three times, and had borne five children by her first husband, but none since. Previously to the last two years, she had suffered but little at her menstrual periods; but since then the pain at such times had been intense, and, not unfrequently, shreds of membrane had been expelled. The second night after her third marriage, about three months before the above date, she was attacked immediately after sexual intercourse, with a profuse hæmoptysis; and this had repeatedly occurred upon any great effort, and generally during or immediately after connection. Had never raised blood before; never had suffered from pulmonary disease, and did not belong to a phthisical family.

For a few weeks past she had perceived a sensible swelling in the abdomen; thought herself to be pregnant, and insisted that she could distinguish the motions of the child, and that her sensations were precisely such as she had always experienced when in this situation. The catamenia, though diminished, occurred at each regular period. An examination of the abdomen was made through the dress, which, however unsatisfactory, determined me that she was not pregnant; and so I endeavored to persuade her, but could not.

In January, more than six months after I first saw her, I was sent for, and found her in bed; complaining, as she expressed it, of all the symptoms which she formerly experienced, when pregnant, and anxiously expecting to be confined. Her abdomen was much distended; there was slight tenderness on deep pressure, and she still insisted that she felt the motions of the child. Upon examination of the abdomen, which was loaded with fat, I could detect no defined firm tumor; and I could hear no placental murmur, nor the sound of the fœtal heart. The areolæ were darker, she said, than they ever had been in her former pregnancies; and milk was secreted by the breasts. The catamenia had continued to occur regularly since June, though scanty, and with great distress. Upon examination per vaginam, I found the neck of the uterus not obliterated; the body was enlarged, and yielded upon pressure, as if no solid substance was contained within it. I again told her that she was not pregnant; gave her a drastic purgative, which operated freely, and visited her for a day or two, during which she became more comfortable.

On the 12th of March, supposing herself to be pregnant, I was again sent for. I found it was a menstrual period, and that while she was suffering severe pain, about a quart of orange-colored fluid suddenly flowed from the uterus, and she supposed the membranes had broken. About two gallons of this fluid escaped during the following week; after which it ceased for a week; and then, during a day and night, a quantity passed, which it was supposed would fill an ordinary water-pail. For the last week (April 8th) there has been no flowing. A catamenial period is now present, and the pain she has suffered for two years is absent; she experiences no inconvenience, has diminished much in size, and weighs about thirty pounds less than she did a week ago.—*Ibid.*

